# Renal Recovery after the Implementation of an Electronic Alert and Biomarker-Guided Kidney-Protection Strategy following Major Surgery

**DOI:** 10.3390/jcm10215122

**Published:** 2021-10-31

**Authors:** Laszlo Halmy, Joshua Riedel, Florian Zeman, Birgit Tege, Volker Linder, Carsten Gnewuch, Bernhard M. Graf, Hans J. Schlitt, Tobias Bergler, Ivan Göcze

**Affiliations:** 1 Department of Surgery, University Hospital Regensburg, Franz-Josef-Strauß-Allee 11, 93053 Regensburg, Germany; laszlo.halmy@ukr.de (L.H.); Hans.schlitt@ukr.de (H.J.S.); 2 Medical Faculty, University of Regensburg, Universitätsstraße 31, 93053 Regensburg, Germany; joshua.riedel@t-online.de; 3 Center for Clinical Studies, University Hospital Regensburg, Franz-Josef-Strauß-Allee 11, 93053 Regensburg, Germany; florian.zeman@ukr.de; 4 Department IT, Information Technology and Clinical Applications, University Hospital Regensburg, Franz-Josef-Strauß-Allee 11, 93053 Regensburg, Germany; birgit.tege@ukr.de (B.T.); volker.linder@ukr.de (V.L.); 5 Institute for Clinical Chemistry and Laboratory Medicine, University Hospital Regensburg, Franz-Josef-Strauß-Allee 11, 93053 Regensburg, Germany; carsten.gnewuch@ukr.de; 6 Department of Anesthesiology, University Hospital Regensburg, Franz-Josef-Strauß-Allee 11, 93053 Regensburg, Germany; bernhard.graf@ukr.de; 7 Department of Nephrology, University Hospital Regensburg, Franz-Josef-Strauß-Allee 11, 93053 Regensburg, Germany; tobias.bergler@ukr.de

**Keywords:** AKI, major surgery, electronic alert, biomarker, recovery

## Abstract

**Background:** The facilitation of early recovery of acute kidney injury (AKI) is an important step to improve outcome, particularly because of the limited therapeutic interventions currently available for AKI. The combination of an electronic alert and biomarker-guided kidney-protection strategy implemented in the routine care may have an impact on the incidence of early complete reversal of AKI after major non-cardiac surgery. **Methods:** We studied 294 patients in two cohorts before (*n* = 151) and after protocol implementation (*n* = 143). Data collection required 6 months for each cohort. The kidney-protection protocol included an electronic alert to detect patients who were eligible for urinary biomarker [TIMP2 × IGFBP7]-guided kidney-protection intervention. Intervention was stratified according to three levels of immediate AKI risk: low, moderate, and high. After intervention, postoperative changes in the glomerular filtration rate (eGFR) were identified with a tracking software that included an alert for nephrology consultation if the eGFR had declined by >25% from the preoperative reference value. Primary outcome was early AKI recovery, i.e., the complete reversal of any AKI stage to absence of AKI within the first 7 postoperative days. **Results:** Protocol implementation significantly increased the recovery of AKI (36/46, 78% compared to control 27/48, 56%, (*p* = 0.025)) and reduced the length of the ICU stay (*p* < 0.001). There was no significant difference in the overall incidence of all AKI and moderate and severe AKI in the first 7 postoperative days: 46/143 (32%) and 12/151 (8%) in the protocol implementation group compared to 48/151 (32%) and 18/151 (12%) in the historical control group. Patients with AKI reversal within the first 7 postoperative days had lower in-hospital mortality than patients without AKI reversal. **Conclusions:** Implementing a combined electronic alert and biomarker-guided kidney-protection strategy in routine care improved early recovery of AKI after major surgery.

## 1. Introduction

The second most frequently reported etiology of acute kidney injury (AKI) of intensive care patients is major surgery. Incidence rates of AKI after surgery vary between 6.7% [1] and 39.3% [2], and about 2.3% [3] to 6.8% [4,5] of these patients require renal replacement therapy (RRT). However, despite the growing knowledge in the field of postoperative care, AKI carries the risk of both short-term and long-term mortality [5,6] and causes high costs for public health-care systems [7,8,9]. Recent advances in AKI research have enabled a shift from solely assessing the functional renal markers creatinine and urine output to monitoring changes on the cellular level—subclinical injury—before altering renal function with new biomarkers [10,11,12,13]. Biomarkers of kidney stress (the urine concentration product of the tissue inhibitor of metaloproteinases-2 and insulin-like growth factor-binding protein 7-TIMP2 × IGFBP7) contribute to cell-cycle inhibition and rapidly increase after surgery or nephrotoxin exposure but do not remain in urine once the insult has passed have been validated [14].

Our previous data have shown that the timely implementation of preventive strategies effectively prevents AKI in patients with elevated stress biomarkers after a kidney insult such as major surgery [15]. As a result of the current lack of specific therapeutic interventions for clinically manifest AKI, recovery patterns after AKI have also increasingly become the focus of research [16]. It is unclear if such a kidney-protection strategy for the time course of AKI may also facilitate renal recovery and thus the subsequent clinical course of patients.

Recently developed instruments for possibly increasing the efficacy of preventive measures are automated electronic alerts. Although the implementation of automated, electronic alerts for AKI recognition alone in adults showed conflicting results [17,18], we hypothesized that the implementation of a protocol that combines a computed electronic alert with a biomarker-guided kidney-protection strategy in routine care after major surgery may increase alertness, thereby leading to early reversal of postoperative AKI.

We retrospectively evaluated and compared patient subgroups before and after protocol implementation to determine the superiority of such a recognition and early intervention strategy in patients at risk for AKI development.

## 2. Materials and Methods

### 2.1. Patients and Study Design

This quality improvement initiative retrospectively evaluated patients after major non-cardiac surgery before and after the implementation of an electronic alert and biomarker-guided kidney-protection strategy. Patients eligible for assessment for both cohorts had to be adults who required observation and intensive care after major abdominal or vascular surgery of >4 h and who had intraoperatively received a jugular central venous line and a urinary catheter. Prolonged surgical time (>4 h) is recognized as an intraoperative risk factor for AKI in non-cardiac surgical patients [19]. Moreover, the operative time of >4 h was one of the procedure-related factors that apply for the definition of major surgery. Consequences of major surgery are high morbidity (>30% overall) and mortality (>2%) [20]. The exclusion criteria in our study were preoperative moderate or severe AKI within 7 days before surgery or at admission to the intensive care unit (ICU), pre-existing severe chronic kidney disease (an estimated glomerular filtration rate (GFR) of <15 mL/min), previous renal replacement therapy, history of kidney transplantation, pregnancy, and breastfeeding. AKI was defined according to the KDIGO 2012 guidelines [21]. The study protocol was approved by the local Institutional Review Board (IRB) (Ethics Committee, University of Regensburg, no. 19-1555-101). Requirement of written informed consent was waived by the IRB. Patients in the control cohort had to have received standard intensive care and a surgical procedure of duration >4 h between 1 October 2018 and 31 March 2019. A kidney-protection protocol had to have been implemented as part of the routine care between 1 April 2019 and 25 May 2019. The protocol implementation cohort included all patients who had received surgery between 26 May 2019 and 25 November 2019. There was no other change in the routine medical or surgical treatment of these patients. A flowchart of the study is presented in Supplementary Figure S1.

### 2.2. Electronic Alert and Biomarker-Guided Kidney-Protection Protocol

**Figure 1:**
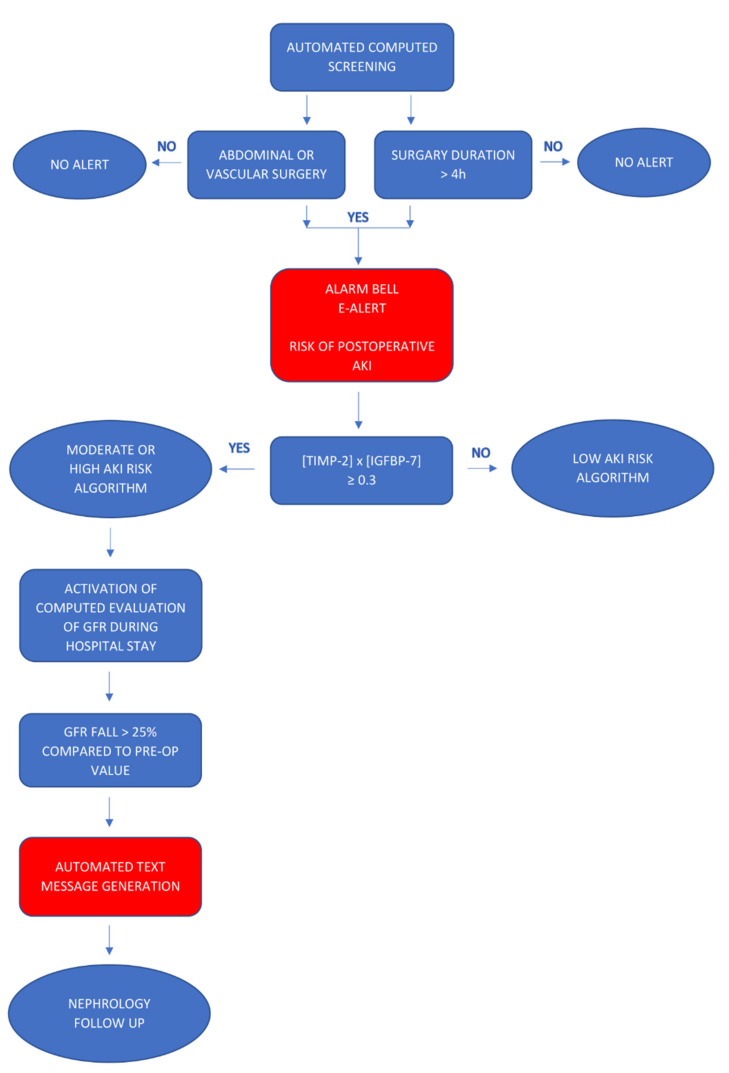
Algorithm for generating an automated electronic alert.

The electronic alert and biomarker-guided kidney-protection strategy included the automatic detection of eligible surgical patients by means of a patient data management system (SAP^®^). The algorithm for electronic alert generation is presented in Figure 1. The duration and type of the surgical procedures were documented during surgery and automatically tracked through a software program. The electronic files of the patients who had received major abdominal or vascular surgery of >4 h and had to be admitted to the ICU were marked by means of a visible electronic “alarm bell” that provided information about the risk of postoperative AKI. After ICU admission, identified patients were tested for levels of urinary TIMP-2 × IGFBP7 using the Astute Medical NephroCheck^®^ test, a point of care unit-use immuno-fluorescence assay on the ASTUTE140^®^ Meter with a 20-minute reaction time. The risk of AKI (TIMP-2 *×* IGFBP7) was derived from (cTIMP-2 *×* IGFBP7)/1000 with a 0.3 (ng/mL) 2/1000 cut-off. Results were automatically transferred from the database of the hospital laboratory to the electronic patient file. The ICU team was informed by phone to ensure immediate intervention. Three levels of AKI risk were established according to recent biomarker data: low risk with TIMP-2 × IGFBP7 < 0.3; moderate risk TIMP-22 × IGFBP7 0.3–2.0, and high risk TIMP-2 *×* IGFBP7 > 2.0 [22]. In patients with a low risk, fast-track step down was possible with early discharge from the ICU, if not indicated otherwise. Repetition of TIMP-2 × IGFBP7 was only recommended in the case of changes in the clinical condition within the first 24 h after surgery. Recommendations for patients with a moderate risk included daily creatinine measurements, hourly monitoring of urine output, and avoidance of nephrotoxic medication. Volume status was assessed with static or dynamic tests such as central venous pressure (CVP) measurement, echocardiography, sonography of inferior vena cava (IVC), or the leg-raising test. Adequate perfusion pressure was maintained with fluid therapy or vasopressors. Patients with a high risk required additional nephrology consultation, maintenance of mean arterial pressure within a 10% margin of preoperative values, and extended hemodynamic monitoring.

**Figure 2:**
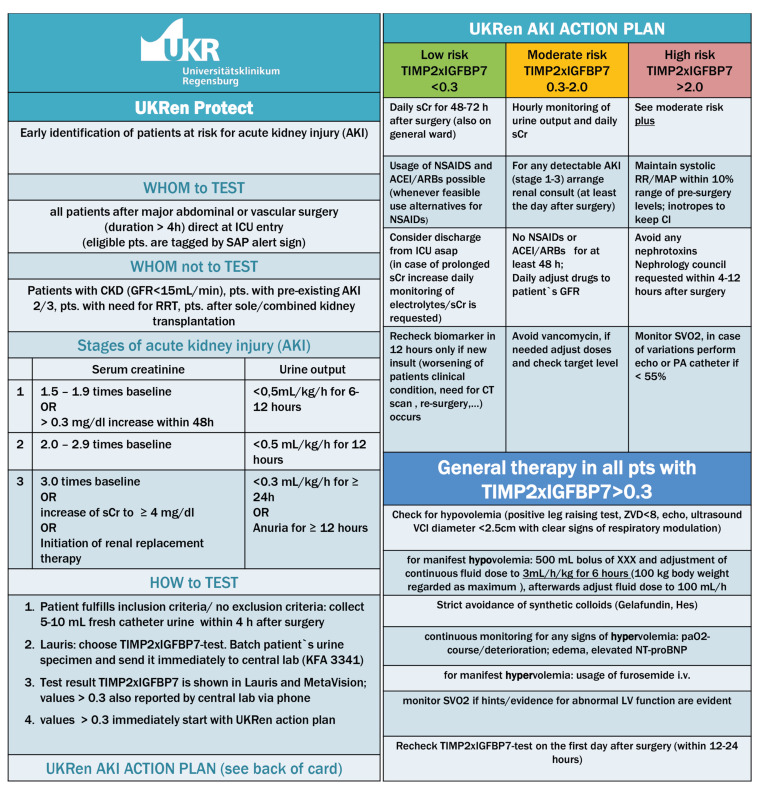
The renal intervention recommendations.

The intervention recommendations are summarized in Figure 2. These recommendations were accessible as a PDF file in the electronic patient records at the bedside, and the ICU team had been informed and trained before protocol implementation. The intervention recommendations in the present study were based on our previous work and on the also recently published recommendations of an expert panel for the clinical use of TIMP-2 × IGFBP7 biomarker testing to assess the risk of AKI [23].

Finally, all patients who were marked by an “alarm bell” in the patient data management system and had at least a moderate risk of AKI were screened for changes in the estimated glomerular filtration rate (eGFR) during the entire postoperative period. For the estimation of GFR, the CKD-EPI formula was used. If a decrease in the eGFR > 25% of the preoperative baseline values was detected by means of the tracking system, an automated message was sent through the in-hospital electronic mailing system with an alert for nephrology follow-up. The standardized alert text contained the patient ID, location in the hospital, eGFR before surgery, last eGFR, and the drop in the eGFR as percentage. Patients with decreased eGFR on the general ward received a nephrology follow-up, whereas nephrologic consultation on ICU was limited to patients with a combination of eGFR decline and a highly elevated kidney stress biomarker (TIMP-2 *×* IGFBP7 > 2.0). The chosen eGFR decline > 25% is included in RIFLE classification of AKI and has been validated as a good outcome predictor [24].

### 2.3. Outcomes

The primary outcome was to determine whether protocol implementation may facilitate early AKI recovery defined as a complete reversal of any AKI stage to absence of AKI within the first 7 days after surgery.

The secondary endpoints included the time to AKI, the time to moderate or severe AKI, the incidence of AKI, the incidence of moderate or severe AKI, and the need of renal replacement therapy during the first 7 days after surgery. Additionally, we analyzed the length of ICU and hospital stays and overall in-hospital mortality. The mortality of patients with early reversed AKI was compared with that of patients with persistent AKI over the first 7 days after surgery. Although illness severity did not differ between the two cohorts, we adjusted the outcomes for heart failure, pre-existing chronic kidney disease (eGFR < 60 mL/min), and peripheral vascular disease. The reason for this adjustment was that these comorbidities showed different frequencies between the two cohorts and are classified under susceptibilities for AKI in the KDIGO guidelines [25]. Serum creatinine was measured before surgery, at ICU admission, on a daily basis during the ICU stay, and as indicated by the responsible physician during the stay at the general ward. Urine output was assessed hourly during the ICU stay.

### 2.4. Statistical Analyses

No a priori sample size calculation was performed, since there were no assumptions about effect sizes, which could be expected. Nevertheless, we estimated to recruit about 150 patients within 6 months, which is sufficient enough to get precise effect estimates with enough power for detection of small to medium effect sizes.

Data are presented as absolute and relative frequencies (%) for categorical variables and as median (interquartile range (IQR)) for continuous data. Categorical variables were compared between both groups by using the Chi-Square Test of Independence, and continuous data were compared by using the Mann–Whitney U-test. The primary endpoint and all secondary endpoints are also presented as odds ratio (OR) (95% confidence interval) for binary outcomes and as Cliff’s Delta (95% confidence interval) for continuous outcomes. The primary endpoint was further analyzed by using a multivariable logistic regression model, which was adjusted for the known confounders heart failure, chronic kidney disease, and peripheral vascular disease. Secondary endpoints with binary outcomes were analyzed analogous to the primary endpoint. The level of significance was set to α = 5% for the primary and for all secondary endpoints. Since secondary endpoints are of an exploratory nature, no adjustment for multiple testing was performed. All analyses were performed using R, version 4.0.1 (The R Foundation for Statistical Computing, Vienna, Austria).

## 3. Results

### 3.1. Patient Characteristics

Overall, 294 patients fulfilled the inclusion criteria for final evaluation. The protocol implementation cohort (*n* = 143) was compared with the historical control cohort (*n* = 151). Patients in the protocol implementation group showed a higher percentage of chronic heart failure (24% vs. 11%, *p* = 0.003) and chronic kidney disease (26% vs. 13%, *p* = 0.004) but a lower percentage of peripheral arterial disease (6% vs. 16%, *p* = 0.009). The remaining baseline characteristics of the study participants including age, BMI, preoperative kidney function, and comorbidities were well balanced between the two groups (Table 1). The two cohorts showed similar indications for surgical procedures most often consisting of hepatobiliary, vascular, or colorectal surgery, duration of intraoperative anesthesia, duration of the surgical procedure, preoperative ASA risk classification, and severity of illness score SAPS II at ICU admission. The perioperative characteristics are shown in Table 2. In the protocol implementation cohort, [TIMP2 × IGFBP7] was measured in all patients at ICU admission: 46 (32.2%) patients had a low AKI risk (TIMP2 × IGFBP7 < 0.3), 81 (56.6%) had a moderate AKI risk (TIMP2 × IGFBP7 0.3–2.0), and 16 patients (11.2%) had a high AKI risk (TIMP2 × IGFBP7 > 2). Median postoperative TIMP2 × IGFBP7 was 0.46 (Q1, Q3 0.23, 0.96).

### 3.2. Clinical Outcomes

#### 3.2.1. Primary Endpoint

**Figure 3:**
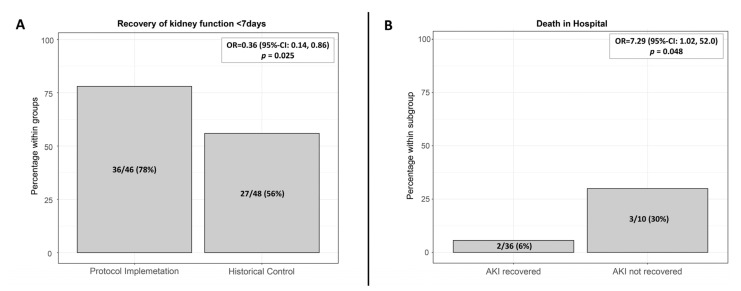
(**A**) Recovery of renal function within the first 7 days after surgery in the historical control group and after protocol implementation, *p =* 0.025 (Chi-Quadrat-Test); (**B**) Protocol implementation cohort: In-hospital mortality of patients with complete AKI recovery within the first 7 days after surgery (2/36), of patients without recovery (3/10), *p =* 0.048. AKI: Acute kidney injury.

Overall incidence of AKI in the first 7 postoperative days was 46/143 (32%) in the protocol implementation group compared to 48/151 (32%) in the historical control group. AKI recovery, defined as the complete reversal of AKI within the first 7 days after major surgery, occurred in 36/46 (78%) patients in the protocol implementation cohort compared to 27/48 (56%) patients in the historical control group (OR; 0.36 (95% CI: 0.14, 0.86), *p* = 0.025) (Figure 3A). After adjustment for the comorbidities heart failure, chronic kidney disease, and peripheral vascular disease, the occurrence of AKI recovery was still significantly higher in the protocol implementation cohort (OR; 0.35 (0.13, 0.90), *p* = 0.029) than in the historical control group.

#### 3.2.2. Secondary Endpoints

In the protocol implementation cohort, mean time of developing postoperative AKI was 1.42 (SD 1.5) days and of moderate or severe AKI was 2.82 (SD 1.72) days; the respective figures for the historical control group were 1.35 (SD 1.67) and 2.61 (SD 2.00) days. Patients without AKI showed significantly lower in-hospital mortality than patients with AKI: 1% (1/97) vs. 11% (5/46); OR 11.7 (95% CI: 1.33, 103), *p* = 0.027 (Figure 3). There was no significant difference in the overall incidence of all AKI and moderate and severe AKI in the first 7 postoperative days 46/143 (32%) and 12/151 (8%) in the protocol implementation group compared to 48/151 (32%) and 18/151 (12%) in the historical control group, although secondary outcomes tended to be consistently better in the protocol implementation group.

Protocol implementation was associated with more RRT-free days (mean 6.97 (SD 0.46) vs. 6.33 (1.7), *p* = 0.027), and shorter ICU stays (median 3 (IQR 1, 4.5) days vs. 3 (2, 6) days, *p* < 0.001) than in the historical control group. The ICU readmission rate was similar in both cohorts 30 (21%) vs. 25 (17%), *p* = 0.331. All outcome endpoints are summarized in Table 3 and Table 4.

In the low-risk AKI subgroup (TIMP2 × IGFBP7 < 0.3), 14/46 patients developed AKI. The recovery rate within the first 7 days after application of the low-risk AKI algorithm was 78% (11/14). In the moderate and high AKI risk subgroup, 32/97 of patients developed AKI. A similar recovery rate of 79% (25/32) was achieved through the tailored application of moderate or high-risk AKI algorithm and additional nephrology follow-up, if eGFR declined > 25%. Patients with AKI recovery had a lower in-hospital mortality rate than patients with persistent AKI at day 7 after surgery: 5.6% (2/36) vs. 30% (3/10), OR 7.29 (95% CI: 1.02, 52), *p* = 0.048 (Figure 3B).

Overall, 93 electronic alerts with an automated massage were generated in the protocol implementation group for 35 patients who had shown a decrease in the eGFR of more than 25% after intervention. For seven patients, the first postoperative drop in the eGFR had occurred at the general ward, and for 28 patients, it occurred during the postoperative ICU stay. All general ward AKI patients who received a nephrologic consultation displayed full recovery of AKI within 14 days after surgery.

## 4. Discussion

This retrospective evaluation included patients who had been admitted to intensive care unit after major surgery and compared two cohorts before (historical control) and after the implementation of a combined electronic alert and biomarker-guided kidney-protection strategy in routine postoperative care over 6 months. The kidney protection protocol included an electronic alert for the detection of eligible patients that triggered urinary biomarker [TIMP2 × IGFBP7]-guided intervention with three risk levels—low, moderate and high—according to TIMP-2 × IGFBP7 values. Additionally computed tracking of postoperative eGFR changes for patients with at least moderate AKI levels with alert for nephrology follow-up if eGFR declined > 25% in postoperative course was implemented. Protocol implementation was associated with a higher rate of AKI recovery, more RRT-free days, and shorter ICU stays.

The strength of our protocol is the fact that the first two steps—alert for detecting eligible patients and biomarker-guided intervention—took place before AKI became evident. In previous studies, electronic AKI alerts had not been activated prior to the alteration in kidney function [26]. However, for adequate AKI prevention strategies, patients at risk of AKI have to be identified before the manifestation of kidney damage. A recent consensus paper described the role of validated biomarkers to identify patient populations for whom preventive interventions have been shown to improve outcomes [23,27]. In addition to the introduction of alert systems in early AKI detection and biomarker-driven AKI identification, automated systems are the object of scientific debate. Recently, Ugwuowo et al. [28] published an AKI real-time prediction algorithm in which only hyaline casts on a urine microscope and fractional excretion of urea nitrogen differed between patients who developed AKI within 24 h and patients who did not. However, investigated patients in this study and ours differ in several aspects, and biomarkers have been measured in the other trial only in a small proportion of patients. Park et al. reported a change in clinicians’ behavior after the implementation of an AKI alert system that automatically generates nephrology consultation. Alert systems reduced the number of overlooked AKI events and increased the odds for early nephrology consultation [29].

Recently, reduced AKI incidence was shown for post-cardiac surgery patients [30] as well as patients after non-cardiac major surgery [15] when being identified as high risk by biomarker testing and randomized prior to KDIGO bundle treatment. To our knowledge, the effects of a combined biomarker-guided kidney-intervention and electronic alert on AKI recovery have neither been established nor investigated so far. The achieved effects in our study may be due to the combination of improved efficacy in detecting patients at risk by means of an automated electronic alert and early stratification according to the respective kidney stress followed by targeted renal intervention. Second alerts tracked a decrease in kidney function despite intervention and initiated early nephrology follow-up, which ensured precise nephrologic care also for patients after ICU discharge on the general ward. Several clinical trials have shown an improved outcome of patients with AKI reversal. Recently, a retrospective single-center analysis of intensive-care patients characterized several AKI recovery patterns and related them to a 1-year outcome. Early reversal within 7 days of AKI occurred in 63% of patients and was associated with improved outcome [31]. A study of AKI after major abdominal surgery reported an incidence of 22% of postoperative AKI, a recovery rate of 52% in the first 48 h, and increased mortality of 52% in patients with persistent AKI [32]. The AKI recovery rate of 78% found in our study was higher than previously reported and was associated with significantly lower in-hospital mortality compared to patients with persistent AKI after the first 7 days. However, early reversal may still be associated with an increased risk of chronic kidney disease 1 year after hospitalization [33]. AKI that persists longer than 7 days after exposure is defined as an acute kidney disease (AKD), which passes into CKD after duration of >90 days [34]. Continuous extension of AKI to AKD and CKD depends on the capability of a damaged kidney to regenerate its normal architecture or whether the regeneration process becomes maladaptive. One of the drivers for maladaptive repair and thereby enhanced progression to CKD is the cell cycle arrest of tubular cells [35]. Correspondingly, studies suggested a strong association between the detection of cell cycle arrest biomarkers TIMP2 × IGFBP7 and adverse outcome—loss of kidney function and mortality [36,37]. Therefore, the AKI prevention strategies in our study were stratified according to biomarker levels with more complex measures and nephrology involvement in subgroups with elevated biomarker levels (TIMP2 × IGFBP7 ≥ 0.3) and a lower degree of intervention with fast-track ICU discharge pathway in the subgroup with negative biomarkers (TIMP2 × IGFBP7 < 0.3). This algorithm finally led to a similar degree of AKI recovery in both subgroups despite substantial differences in AKI risk profile.

In the current study, the alert and kidney-protection strategy was implemented in a patient population for whom AKI constitutes a significant complication with a major impact on outcome. The influence of postoperative AKI on higher in-hospital mortality has been demonstrated in patients after major abdominal surgery. In a systematic meta-analysis of 82,514 patients, O’Connor reported a 12.6-fold relative mortality risk in patients with postoperative AKI [38]. Similarly, the development of AKI was associated with a 11.7-fold higher rate of in-hospital mortality in our study.

The effects on the incidence of AKI in the current study were less obvious than in our previous biomarker implementation interventional randomized BigpAK trial [15]. Protocol implementation over 6 months as in the current study was associated with a reduction in moderate and severe AKI of 33.3% compared to 66.0% in the BigpAK study. However, there are some significant differences between the two studies. Current results were achieved in daily routine and included every patient who had been transferred to the ICU after major non-cardiac major surgery, where a significant proportion of these patients (32.2%) had only a low risk of AKI. In the BigpAK trial, only patients with a moderate or high risk of AKI (TIMP2 × IGFBP7 > 0.3) were randomized for intervention. This difference is reflected by the fact that values of tubular cellular stress (median TIMP2 × IGFBP7) at ICU admission were higher (0.96) in the BigpAK trial than in the current study (0.46). Additionally, patients in the current protocol implementation group had been significantly more often affected by CHF and CKD—two well-known risk factors for subsequent AKI [39].

The study has some major limitations. Retrospective and mono-centric design does not allow for any generalization of the findings. The study investigated neither the trajectories of renal recovery nor the quality performance of the used alerts, because these factors have been extensively investigated and reported in other studies. Another important point to note is that the study was not designed to analyze if usage of the combination of electronic alerts with the measurement of biomarkers is superior to the application of either single approach. Finally, the study did not investigate the long-term effects of the intervention. The results provide first data on the relevance of a combination of electronic alert and biomarker-guided targeted AKI intervention in clinical routine care. This combination may mitigate the magnitude of perioperative kidney damage and improve early recovery. The current study provides pilot data that need to be confirmed in an adequately powered prospective study.

## Figures and Tables

**Table 1:** Baseline characteristics.

	Protocol Implementation *n* = 143		Historical Control *n* = 151		*p*-Value
Age, median (IQR)	64 (55–74)		65 (56–73)		0.85
Sex (%)	Female	Male	Female	Male	
	58 (41%)	85 (59%)	45 (30%)	106 (70%)	0.053
BMI, median (IQR)	25 (23–29)		26 (23–29.5)		0.365
Weight, kg, median (IQR)	76 (65–86)		80 (69.5–90)		0.089
SAPS II, median (IQR)	30 (23–38.5)		30 (24.25–37.75)		0.956
Preoperative creatinine, mg/dL, median (IQR)	0.84 (0.7–1.02)		0.83 (IQR 0.7–0.97)		0.898
Preoperative eGFR (CKD-EPI), mL/min/1.73 qm, median (IQR)	86 (71–99)		90 (75–98)		0.424
Comorbidities					
Chronic cardiac disease	24 (17%)		26 (17%)		0.921
Chronic heart failure	35 (24%)		17 (11%)		0.003
Arterial hypertension	70 (49%)		74 (49%)		0.992
Diabetes mellitus	29 (20%)		20 (13%)		0.106
Chronic liver failure	8 (6%)		8 (5%)		0.911
Smoking	16 (11%)		23 (15%)		0.307
Chronic kidney disease eGFR < 60 mL/min	37 (26%)		19 (13%)		0.004
COPD	8 (6%)		6 (4%)		0.514
Peripheral vascular disease	9 (6%)		24 (16%)		0.009
Malignancy	92 (64%)		108 (72%)		0.187

**Table 2:** Surgical characteristics.

	Protocol Implementation *n* = 143	Historical Control *n* = 151	*p*-Value
ASA score preoperative			0.400
ASA 1	8 (6%)	6 (4%)	
ASA 2	45 (32%)	46 (31%)	
ASA 3	85 (59%)	86 (58%)	
ASA 4	5 (3%)	11 (7%)	
Type of surgery			
Hepatobiliary surgery	55 (38%)	53 (35%)	0.550
Transplantation	4 (3%)	2 (1%)	0.449
Pancreatic surgery	21 (15%)	22 (15%)	0.978
Upper-GI surgery	8 (6%)	6 (4%)	0.514
Colorectal surgery	17 (12%)	26 (17%)	0.196
Vascular aortic surgery	11 (8%)	16 (11%)	0.389
Vascular other surgery	9 (6%)	10 (7%)	0.909
Multivisceral resection and HIPEC	12 (8%)	5 (3%)	0.062
Multivisceral resection without HIPEC	8 (6%)	8 (5%)	0.911
Other surgery	0 (0%)	1 (1%)	0.330
Duration			
Duration of surgery (minutes)	295 (IQR 233.5–381)	300 (IQR 219–384.5)	0.741
Duration of anesthesia (minutes)	365 (IQR 310.5–463.5)	380 (IQR 298.5–473)	0.951

**Table 3:** Clinical outcome of protocol implementation group vs. historical control group.

	Protocol Implementation *n* = 143	Historical Control *n* = 151	Effect Estimate (95% CI) *	*p*-Value
Primary outcome				
Recovery of kidney function in 7 days	36/46 (78%)	27/48 (56%)	0.36 (0.14, 0.86)	0.025
Secondary outcomes				
Overall AKI (%) in 7 days	46 (32%)	48 (32%)	0.98 (0.6, 1.61)	0.944
AKI stage 2 and 3 in 7 days	12 (8%)	18 (12%)	1.48 (0.69, 3.26)	0.320
AKI stage 3 and dialysis in 7 days	2 (1%)	7 (5%)	3.43 (0.70, 16.8)	0.129
ICU length of stay, median (IQR) days	3 (1–4.5)	3 (2–6)	0.30 (0.16, 0.42)	<0.001
Hospital length of stay, median (IQR) days	14 (11–22)	13 (9–18.5)	−0.12 (−0.24, 0.02)	0.083
RRT-free days (SD) in 7 days **	6.91 (0.46)	6.33 (1.7)	−0.58 (−1.09, −0.07)	0.027
In-hospital mortality	6 (4%)	10 (7%)	1.62 (0.59, 4.87)	0.363
Any AKI > 7 days	6 (4%)	9 (6%)	1.45 (0.51,4.42)	0.494
AKI stage 2 and 3 (%) > 7 days	2 (1%)	4 (3%)	1.92 (0.37,13.99)	0.456
Any in-hospital AKI (%)	48 (34%)	57 (38%)	1.20 (0.74, 1.94)	0.455
Any in-hospital AKI stage 2 and 3 (%)	14 (10%)	22 (15%)	1.57 (0.77, 3.21)	0.214

* Odds ratio (95%-CI) for binary outcomes and Cliff’s Delta for continuous outcomes; ** only patients with AKI (*n* = 46 and *n* = 48).

**Table 4:** Clinical outcome of protocol implementation group vs. historical control group (multivariable-adjusted *).

	Odds Ratio (95%-CI)	*p*-Value
Primary outcome		
Recovery of kidney function in 7 days	0.35 (0.13, 0.90)	0.029
Secondary Outcomes		
Overall AKI in 7 days	1.11 (0.66, 1.87)	0.697
AKI stage 2 and 3 in 7 days	1.79 (0.78, 4.07)	0.167
AKI stage 3 and dialysis in 7 days	3.60 (0.62, 20.84)	0.153
In-hospital mortality	2.22 (0.70, 7.00)	0.175
Any AKI stage > 7 days	1.68 (0.54, 5.25)	0.370
AKI stage 2 and 3 > 7 days	1.94 (0.29, 13.03)	0.497
Total in-hospital AKI	1.32 (0.80, 2.20)	0.283
Total in-hospital AKI stage 2 and 3	2.08 (0.95, 4.54)	0.066

* The logistic regression model of each endpoint was adjusted for heart failure, chronic kidney disease, and peripheral vascular disease.

## Data Availability

Data available on request due to restrictions e.g., privacy or ethical. The data presented in this study are available on request from the corresponding author. The data are not publicly available due to privacy police.

## Supplementary Materials

The following are available online at www.mdpi.com/article/10.3390/jcm10215122/s1, Figure S1: Flowchart of the study.
