# Supplementary figures and images for: Renal Recovery after the Implementation of an Electronic Alert and Biomarker-Guided Kidney-Protection Strategy following Major Surgery
Source: J Clin Med. 2021 Oct 31;10(21):5122. doi: 10.3390/jcm10215122 (PMC8584790; doi:10.3390/jcm10215122)

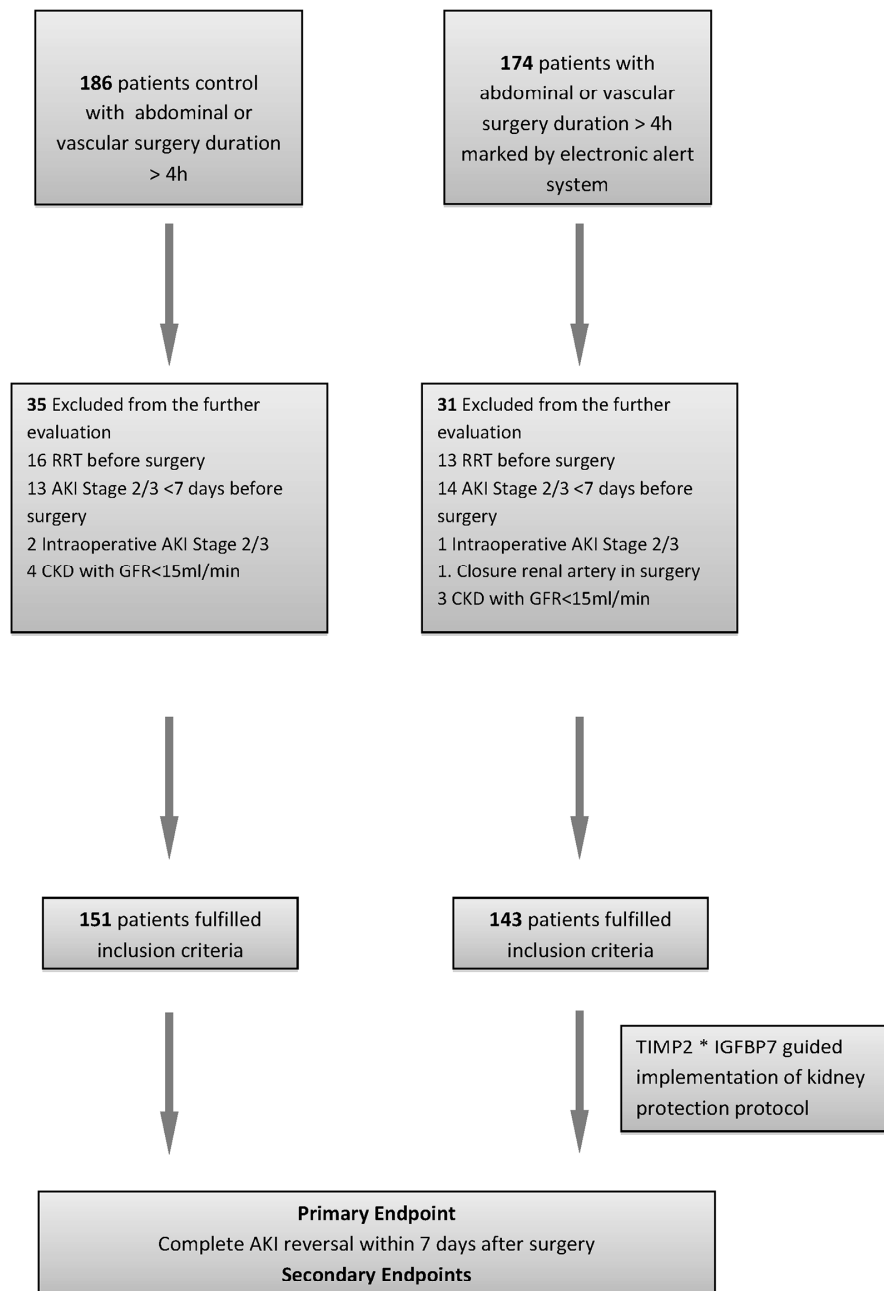

**Figure S1.** Flowchart of the study.

Supplement: Supplementary file 1 [file jcm-10-05122-s001.zip › jcm-1389425-supplementary.pdf]
